# Localized photovoltaic investigations on organic semiconductors and bulk heterojunction solar cells

**DOI:** 10.1088/1468-6996/15/5/054201

**Published:** 2014-10-31

**Authors:** Jan Philipp Kollender, Jacek Gasiorowski, Niyazi Serdar Sariciftci, Andrei Ionut Mardare, Achim Walter Hassel

**Affiliations:** 1 Institute for Chemical Technologies of Inorganic Materials, Johannes Kepler University of Linz, Austria; 2 Christian Doppler Laboratory for Combinatorial Oxide Chemistry (COMBOX) at the Institute for Chemical Technologies of Inorganic Materials, Johannes Kepler University of Linz, Austria; 3 Linz Institute for Organic Solar Cells (LIOS), Physical Chemistry, Johannes Kepler University of Linz, Austria

**Keywords:** photoelectrochemistry, organic semiconductor, bulk heterojunction, scanning droplet cell microscopy

## Abstract

Newly synthesized organic electronics materials are often available in submicrogram amounts only. Photoelectrochemical scanning droplet cell microscopy is a powerful method that allows a comprehensive characterisation of such small amounts including oxidation, reduction potentials, doping, determination of charge carriers, band gap, charge capacity, over-oxidation sensitivity and many more. Localized photoelectrochemical characterization of the poly[4,8-bis-substituted-benzo[1,2-b:4,5-b0]dithiophene-2,6-diyl-alt-4-substituted-thieno [3,4-b] thiophene-2,6-diyl] (PBDTTT-c) and PBDTTT-c:PCBM bulk heterojunction was performed using photoelectrochemical scanning droplet cell microscopy (PE-SDCM). The optical properties and the real and imaginary part of the dielectric function, of the polymer were determined using spectroscopic ellipsometry. The photoelectrochemical characterizations were performed in a three and two electrode configuration of PE-SDCM under laser and white light illumination. The effect of illumination was characterized using dark/illumination sequences. The stability of the photocurrent was studied using longer term (600 s) illumination. Finally the effect of cell configuration and illumination conditions on the photovoltage was studied.

## 1. Introduction

Organic semiconductors were developed in recent years especially due to their possible applications as cheap and environmentally friendly materials for organic electronics. Since their morphological [1] and photophysical [2] properties can be easily modified, a great effort is made for the synthesis and characterization of new organic materials, small molecules [3] and polymers [4]. Also their mechanical flexibility is a big advantage [5, 6]. Most of the polymeric, semiconducting materials used in optoelectronics are derivatives of poly(phenylene vinylene)s, poly(phenylene ethynylene)s and polythiophenes [7]. Recently, new low-band gap materials with broad absorption spectrum ranging from ultraviolet to near infrared, together with good electrical properties, were enhancing the efficiency of the organic solar cells [8, 9]. From many new polymers, poly[4,8-bis-substituted-benzo[1,2-b:4,5-b0]dithiophene-2,6-diyl-alt-4-substituted-thieno[3, 4-b] thiophene-2,6-diyl] (PBDTTT-c) is one of the first synthesized as a highly promising candidate for a new generation of organic semiconductors [10–12]. Due to its high absorption in the visible range and air stability this polymer was successfully used as a donor material in organic solar cells reaching nearly 7% efficiency [13–16] and in photodetectors [17].

Scanning droplet cell microscopy (SDCM) was developed by Hassel and Lohrengel in 1997 for electrochemical investigation of thin aluminium films [18]. The principle behind SDCM is to address only a small part rather than immerse the complete working electrode into the electrolyte like in conventional electrochemical cells. This strong localisation is achieved by bringing a small droplet, formed at the tip of a capillary, in contact with the working electrode (free droplet mode) or by pressing a silicone-sealing-terminated glass capillary against the working electrode (contact mode) [19]. The SDCM in the contact mode is especially important when the electrochemistry on organic materials is performed using non-aqueous electrolytes. Essential is, that this confinement prevents contamination of the electrolyte and ensures reproducibility of the addressed area. By bringing an optical fibre into SDCM (PE-SDCM) all common photoelectrochemical experiments become possible on spot diameters of less than 100 *μ*m. Due to the strong miniaturisation of the addressed area by PE-SDCM the amount of the required material for measurements is drastically reduced (≪1 mg). In this work PE-SDCM was used for local photoelectrochemical characterisation of PBDTTT-c and bulk heterojunction PBDTTT-c:PCBM. Experiments were performed using 635 nm laser or white light illumination.

## 2. Experimental details

For photoelectrochemical studies PBDTTT-c purchased from Solarmer Inc. USA was used as received. The polymer was dissolved in chlorobenzene (99+%, Acros Organics) with a concentration of 20 g L^−1^. For comparison a mixture of PBDTTT-c with (6,6)-phenyl-C_61_-butyric acid methyl ester (PCBM) (Solenne Inc., Groningen, The Netherlands) was prepared. This soluble derivative of buckminsterfullerene was chosen due to its good electron accepting properties. The solution was prepared by dissolving PBDTTT-c and PCBM in chlorobenzene with a total concentration of 20 g L^−1^ and mixing ratio of 1:1 w/w. At a later stage both solutions were spin-cast on two separate 15 × 15 mm^2^ glass/indium tin oxide (ITO, 15 *Ω*/□, Kintec Co.) substrates, and films were formed on the surface after drying in air. The glass/ITO substrates were pre-cleaned by sequential sonication in acetone, isopropanol and de-ionized water and dried in nitrogen flow. The thickness of the PBDTTT-c and PBDTTT-c:PCBM layers was determined by contact profilometry (DekTak XT Stylus profilometer) as 100 and 150 nm, respectively.

The optical properties of the PBDTTT-c polymer were investigated using spectroscopic ellipsometry (SE) from the near-infrared to UV range. The measurements were conducted using a Woollam M-2000 ellipsometer (rotating compensator, Lincoln, USA) which spans an energy range of 0.73 to 6.5 eV. The ellipsometric data was analyzed using specialized software (WVASE™).

**Figure 1.**
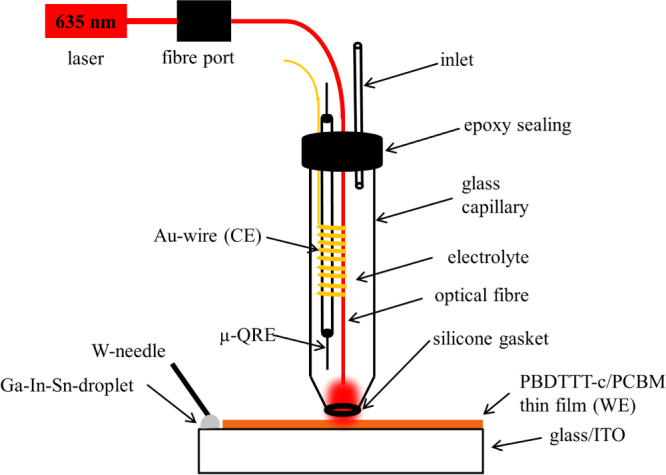
Scheme of the photoelectrochemical scanning droplet cell microscope (PE-SDCM).

All photoelectrochemical measurements presented in this work were conducted using a modified version of a photoelectrochemical scanning droplet cell microscope (PE-SDCM) adapted for organic based electrolytes [20, 21]. The outer part of PE-SDCM was made from a 2.5 mm outer diameter boron silicate glass capillary. The tip was pre-formed using a capillary puller (PC-10, Narishige, Japan) and finished using a home-built polishing machine. To ensure high reproducibility of the addressed area in contact mode a silicone sealing was formed at the rim of the tip by dipping it into liquid silicone and drying it under constant nitrogen flow for several hours. A Ag/AgCl micro-quasi reference electrode (*μ*-QRE) was used. The reference electrode was prepared by electrodepositing AgCl on the first 10 mm of a longer high purity Ag-wire (100 *μ*m diameter) in 1 M HCl [22]. For final assembly of the *μ*-QRE the previously prepared Ag-wire was inserted into a small glass capillary leaving only the AgCl covered part out. Both endings of the small glass capillary were sealed using two component epoxy resin to prevent electrolyte leakage into the reference electrode. The potential of the *μ*-QRE used in this work was measured as 0.211 V vs standard hydrogen electrode (SHE). A spiral-shaped Au wire was used as the counter electrode (CE; 100 *μ*m diameter; 99.999% purity; Wielandt Dentaltechnik, Germany). For final assembly of the PE-SDCM the *μ*-QRE and CE were inserted into the outer glass capillary. Figure 1 shows a scheme of the photo electrochemical scanning droplet cell microscope (PE-SDCM).

To locally illuminate the wetted area a multimode optical fibre with a diameter of 220 *μ*m was additionally inserted into the outer glass capillary. Ends of the optical fibre and the *μ*-QRE were installed in close proximity to the sample surface. As electrolyte a stainless steel syringe with a diameter of 800 *μ*m was installed at the top-part of the PE-SDCM and connected to PTFE tube. As the final fabrication step the top part of the PE-SDCM was sealed using two component epoxy resin. The size of the electrolyte droplet at the tip of the capillary was controlled by means of a high-precision syringe pump.

To determine the area wetted by PE-SDCM anodic oxides were grown on a Ti thin film using an aqueous electrolyte (0.1 M Na_2_SO_4_). High-resolution optical microscopy with automated pattern recognition was used to determine the area of the produced coloured titanium spot [19]. The spot diameter on the WE was 390 *μ*m corresponding to an area of 0.12 *μ*m^2^. Electrical contact of the working electrode (WE) was endured by using a droplet of the ternary Ga-In-Sn eutectic (Alfa Aesar, Germany) on the WE and further contacted by a W needle.

The photoelectrochemical experiments were performed using a 635 nm laser module with auto power control (Roithner Lasertechnik, Austria) and a temperature stabilized tungsten halogen-lamp (HL-2000, Ocean Optics). Both light sources were coupled into an optical fibre using an in-house-developed fibre port. The illumination was controlled by a manually operated shutter. All measurements were performed in a specifically designed dark room to avoid light contamination during the experiments. To measure the optical power density on the addressed area of the WE a fully assembled droplet cell was placed on the detector of an optical power meter (Coherent Lasermate Q, VL54). The measured optical power density was 113 mW cm^−2^ for the red 635 nm laser and 56 mW cm^−2^ for the white light. When using the white light, only a direct determination of the photocurrent at full spectra was performed since for a proper quantum efficiency measurement the generated photocurrents are at the lowest detection limit of the potentiostat used, due to the small irradiated area. During the photoelectrochemical studies presented in this work, both samples were investigated in contact with 0.1 M solution of tetrabutylammonium hexafluorophosphate (TBAPF_6_, ≥99%, Fluka Analytical, Germany), prepared by dissolution in propylene carbonate (PC, 99.7%, Sigma Aldrich, Germany). The electrochemical stability of the electrolyte used was previously checked in a glovebox revealing an electrochemical window between −1.9 and 1.9 V vs Ag/AgCl QRE. Possible air/water contaminations were taken into consideration according to previous studies [23]. To position the tip of the PE-SDCM on the sample surface a gantry robot built from three linear stages was used. To ensure high reproducibility of the addressed area the applied force used to press the tip of the PE-SDCM against the working electrode was continuously monitored using a force sensor (ME-Messtechnik, Germany) and readjusted if necessary by feedback to the *z*-axis. The complete setup was controlled by an in-house developed LabView program. All electrochemical measurements were carried out using a CompactStat Potentiostat (Ivium Technologies, The Netherlands). All measurements were performed using both two and three electrode configurations. The two electrode system is largely used in device characterization but does not allow reliable electrochemical investigations. In the present study results obtained from both systems are shown for comparison.

## 3. Results and discussion

**Figure 2.**
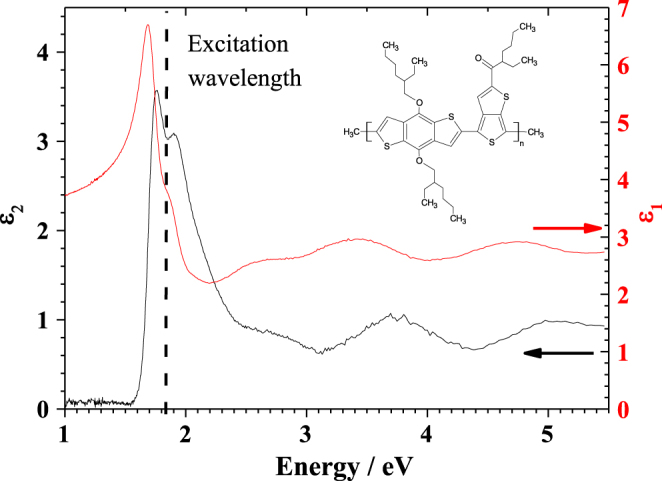
Ellipsometric characterization of the optical properties of the PBDTTT-c (structure in the inset). The real part of dielectric function is plotted in red and imaginary part in black. The dashed line shows the position of the excitation wavelength of the laser used in the photoelectrochemical experiments.

The optical properties of the PBDTTT-c polymer described by the complex dielectric function were characterized using spectroscopic ellipsometry (SE). In this technique the changes in the polarization of the reflected light is measured. According to this, the ratio of the complex Fresnel reflection coefficients for p- (*r*_p_) and s- (*r*_s_) polarized light is determined using the equation1:

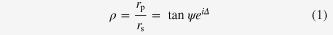
The complex reflection ratio *ρ* is defined by tan*ψ* which represents the amplitude ratio after reflection while *Δ* describes the phase difference between p- and s-polarized light. Using the SE method the values of the *ψ* and *Δ* are directly obtained. At a later stage, using these variables in computer modelling, the real and imaginary part of the dielectric function 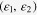 of the material can be determined [24–27]. Prior to the photoelectrochemical studies on PBDTTT-c, the real and imaginary part of the dielectric function of the polymer were characterized using SE. The structure of the polymer is presented in the inset of figure 2. The polymer was synthesized by copolymerization of benzothiophene and bithiophene units. The spin-cast layer was measured at different incidence angles. The resulting ratio *ρ* was fitted to resolve the real and imaginary parts of the dielectric function which are presented in figure 2. The absorption of the polymer is described by the imaginary part of the dielectric function. The plot of 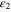 (in black) contains a peak between 1.6 and 2.5 eV constructed from two sharper peaks with maxima at 1.7 and 2.0 eV. This pair of sharp peaks could be attributed to the absorption of benzothiophene and bithiophene units present in the polymer structure. At higher energies (>2.5 eV), broad peaks with maxima at 2.7, 3.8 and 5.0 eV can be found. Their existence can be explained by higher optical transitions in the polymer. The optical energy gap was found at approximately 1.6 eV. The measured optical absorption peaks can be found also in the plot of the real part of the dielectric function according to the Kramers–Kronig relations. In the plot of 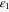 (in red) the presence of two oscillators, with maxima at 1.6 and 1.85 eV, connected with the main absorption peak can be observed. At high energies above the resonance energy, (>2.5 eV) the real part of the dielectric function shows the presence of additional oscillators related to the previously discussed absorptions. Due to the position of the absorption peaks, a laser with 635 nm (1.95 eV) was used for single wavelength photoelectrochemical characterization.

Due to its high localization capability, the use of scanning droplet cell microscopy is expected to improve the understanding and predict possible applications in fields such as organic photovoltaics. Usually, characterization of the photovoltaic effect is done using measurements of the pristine photoactive material as well as its mixture with an acceptor material in a bulk heterojunction (BHJ) solar cell. In both cases an exciton is formed after illumination with photon energy higher than the band gap of the photoactive material. As soon as the photoactive material or BHJ is confined between two electrodes, the exciton formed in the photoactive material can travel due to an applied electric field. At the interface with a metal (electrode) or an accepting material (from BHJ) it separates into free carriers which are transported in opposite directions according to their charge. During device construction material mixtures forming BHJs are preferred due to their stronger output photocurrent. Therefore, in the present work the PBDTTT-c polymer, as well as its mixture with PCBM, was characterized photelectrochemically using SDCM.

For investigating the effect of illumination on the PBDTTT-c and PBDTTT-c:PCBM films in contact with 0.1 M TBAPF_6_ dissolved in PC, sequential dark/illumination photocurrent measurements were performed. To better understand the influence of the illumination on the photoelectrochemical processes, the measurements were performed using either monochromatic (matching the polymer absorption maximum) or white light source. Two different approaches were used for the photoelectrochemical characterization. In a first step, a three-electrode configuration was used with the PE-SDCM for investigations under controlled electrochemical potential. Due to the fact that applications of the studied polymer for photovoltaic devices require only a two electrode configuration, in a second step, the same measurements were done using PE-SDCM without using the QRE.

**Figure 3.**
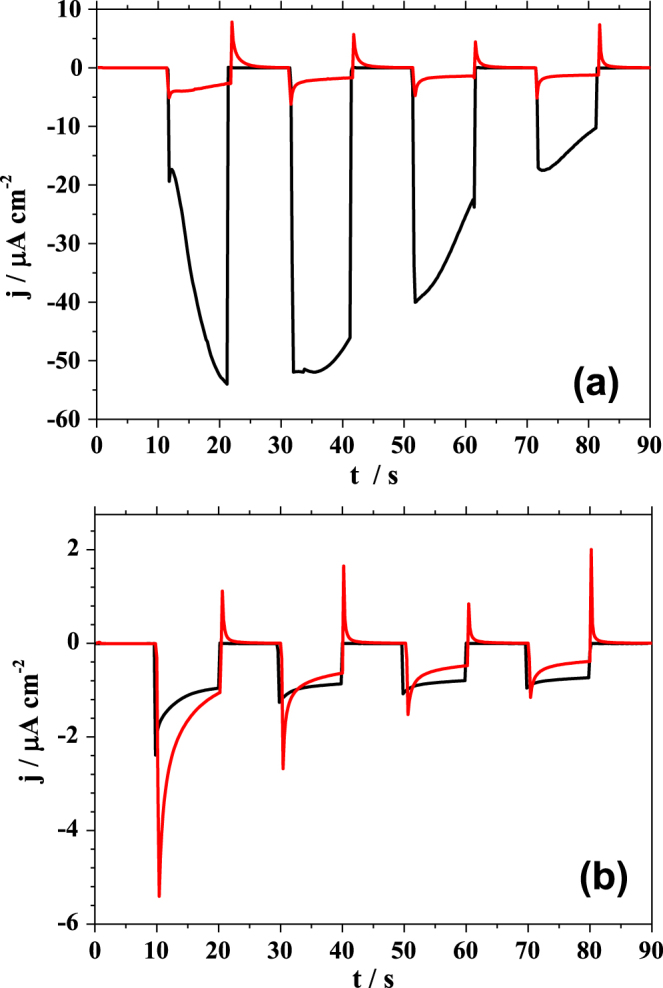
Photocurrent transients measured during illumination with the red laser light (a) and white light (b). The measurement were performed in the three electrode configuration, on pristine PBDTTT-c (black line) and PBDTTT-c:PCBM (red line).

Using the three electrode configuration, the applied potential was set at 0 V vs Ag/AgCl after contacting the surface and the current transients were measured for alternating dark and illuminated conditions. Since at this potential no electrochemical process occurs [27] the measured change in the current can be related only to light induced processes. The resulting current transients are presented in figure 3. In figure 3(a) the dark/illumination sequences as measured using a laser radiation matching the absorption of the polymer (635 nm—see figure 2) are plotted. In the case of PBDTTT-c (plotted in black), during the first 10 s in the dark only the electrochemical background current can be observed. After opening the shutter allowing the radiation to shine through the optical fibre embedded in the SCDM, a strong decrease in the current can be observed. This resulted in a sharp peak with the minimum at −20 *μ*A cm^−2^ recorded within the first second of illumination. This sharp peak is followed by a continuous increase of the measured negative photocurrent down to −55 *μ*A cm^−2^. After 10 s of illumination the shutter was closed and the dark conditions were restored. As a result, the measured current immediately approaches the background electrochemical current level where it remains constant for the next 10 s. After opening the shutter a second time, the current level drops to the value where it was last observed during the first illumination period (−55 *μ*A cm^−2^). After 5 s of radiation exposure, a decrease in the negative photocurrent can be observed. During the third and fourth sequence of illumination with laser light only a smooth decrease of the negative current can be observed. This decrease is a result of the slow degradation of the PBDTTT-c due to the inefficient transport of photogenerated charge carriers from the WE to electrolyte.

The photocurrent transient measured using the PE-SDCM for the PBDTTT-c:PCBM is presented in figure 3(a) in red. During the first 10 s of the experiment only a background current, similar to the one obtained for the pristine polymer, can be seen. After opening the shutter, an interesting situation can be observed when comparing the behaviour of pure polymer under laser illumination with the BHJ. Due to mixing the PBDTTT-c with an accepting unit, an increase of the exciton separation probability (and therefore of the overall charge density) is expected, resulting in a high photocurrent. Surprisingly, the value of the current measured under illumination for the BHJ is much lower as compared to the one measured for the pristine polymer. Also, the shape of the current transient is different. Within the first second of illumination a peak with a minimum at approximately −6 *μ*A cm^−2^ can be noticed. Afterwards the negative current decreases and stabilizes at a level of about −4 *μ*A cm^−2^. Interestingly, upon closing the shutter during the first dark sequence, a positive peak is observed. One possible explanation for this low measured photocurrent could be inefficient charge transport through the BJH leading to charge trapping. However, solar cells based on BHJs sandwiched between metal electrodes exhibit efficiencies above 6% [11]. Also, this theory would not explain the positive peak measured after closing the shutter. However, it can be attributed to discharging the WE/electrolyte interface. The existence of both peaks observed under illumination and in the following dark measurement can be explained by a photodegradation of the BHJ. This process can be associated with charging (under illumination) and discharging (in the dark) of the film. A comparison of the measurements on the film containing PCBM with the pristine material in the photoelectrochemical cell suggests an effect of charging/discharging directly describing the charge trapping in the acceptor. The shape of the discharging and the current flow direction measured in the dark demonstrates the flow of the negative charges towards the working electrode. The next illumination/dark sequences follow the same trend with a slight decrease of the negative photocurrent plateau resulting from a continuation of the degradation process.

Opposite to the expected behaviour of a bulk heterojunction solar cell [28] in the present photoelectrochemical investigation of the PBDTTT-c:PCBM the electrochemical instability of the acceptor strongly hinders the cell performance. The PCBM electrochemical stability plays a crucial role in the charge transport process. While in most of the solid state devices using BHJ containing PCBM the photocurrent is strongly enhanced due to the improved charge extraction [29, 30], in the photoelectrochemical process the acceptor traps most of the charges. In most of the BHJ systems after film formation a phase separation occurs. The interface between the electrolyte and the BHJ can be described either as PCBM/electrolyte or PBDTTT-c/electrolyte. In this situation, a large surface coverage of the PCBM islands [31] would lead to a strong decrease of the photocurrent. Due to this fact, the use of PCBM in contact with 0.1 M TBAPF_6_ in a photoelectrochemical cell is disadvantageous, as opposite to the expectation according to the common knowledge in the field of organic solar cell [29, 30]. Another possible explanation of the low measured photocurrent could be based on the interface recombination where a hole in the PBDTTT-c present at the electrolyte interface recombines with an electron from PCBM.

Due to the fact that the PBDTTT-c was primarily developed to serve as donor material in organic solar cells the photoelectrochemical properties of PBDTTT-c were also assessed under white light illumination. In a manner similar to the laser irradiation experiment, the effect of white light irradiation is reported as a sequence of dark/illumination periods and the resulting photocurrent transients are presented in figure 3(b). Illumination with white light results in a decrease of the overall photocurrent measured for the pristine PBDTTT-c (plotted in black) as compared with the single wavelength case (figure 3(a)). The measured photocurrent is almost 30 times lower as compared to the one measured during laser illumination. One reason for this change is the radiation source power density which in the case of the white light is lowered by a factor of 2. However, it is unlikely that this alone would cause such a strong photocurrent decrease. The shape of the current transients also changed as compared to the case of laser illumination. This time a decrease of the negative photocurrent during the first illumination period is observed until a plateau is reached. The plateau level is reproduced in the next illumination sequence. During laser illumination only the main transition of the polymer is activated (first oxidation step). In this situation, the high instability of the photodoped form of the polymer promotes charge transfer rather than charge trapping. This instability leads to the suppression of the photodegradation process. However, during illumination of the polymer with white light not only the main transition is triggered but also high energy transitions (higher oxidations) can occur. Most likely the stability of these transitions is higher leading to formation of a blocking layer which prevents photogenerated charge flow as suggested by the shape of the current transients plotted in black in figure 3(b). Since the exciton separation can occur only at the polymer/electrolyte interface, the photodegradation dominates the current flow when the pristine PBDTTT-c in contact with 0.1 M TBAPF_6_ dissolved in PC is used.

For analysis of the BHJ where PBDTTT-c is mixed with PCBM, the photocurrent transients measured during white light irradiation are plotted in red in figure 3(b). They show a similar behaviour as compared with the case of single wavelength irradiation. Both, the value and the shape of the current transients for each sequence are weakly dependent on the used irradiation source. The only observed effect of using full-spectral light is evidenced by a slow increase of the photocurrent plateau levels during the light/dark sequences. Different from the case of pristine material, in the BHJ the exciton separation occurs in the bulk instead of electrolyte interface. This is the reason for the observed weak photocurrent dependence on the light source.

Photocurrent measurements using two electrode configuration of the PE-SDCM were performed in order to characterize the photoactive material under typical operation conditions of optoelectronic devices. In these devices the current is measured while a constant potential difference of 0 V is applied between the WE and CE [32]. In the three electrode configuration the potential applied during the photoelectrochemical experiment was set at 0 V in respect to the Ag/AgCl QRE allowing a direct control of the processes occurring at the WE/electrolyte interface.

**Figure 4.**
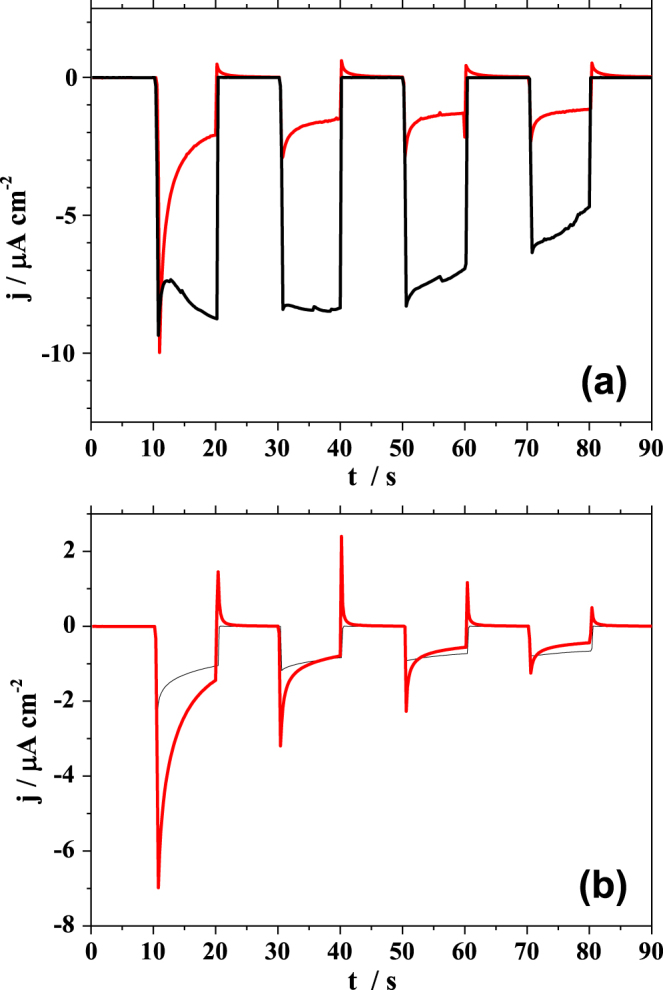
Photocurrent transients measured during illumination with the 635 nm laser light (a) and white light (b). The measurements were performed in the two electrode configuration, on pristine PBDTTT-c (black line) and PBDTTT-c:PCBM (red line).

The photocurrents using PBDTTT-c and PBDTTT-c:PCBM systems were measured during illumination with single wavelength as well as under white light irradiation. The results are presented in figure 4. The photocurrent transients measured using the laser light, were studied and the results are shown in part (a) of figure 4. As compared to the photocurrents measured using the three electrode configuration, the pristine polymer (plotted in black) shows similar shapes of the photocurrent transients. For the first 10 s of the experiment only background current was detected. During the first second of illumination a strong peak with a minimum at −10 *μ*A cm^−2^ can be observed. Its nature can be related to the PBDTTT-c/electrolyte interface charging. Afterwards, a decrease of the negative photocurrent can be observed followed by an increase down to −9 *μ*A cm^−2^. For the next illumination periods, the behaviour of the photocurrent is similar to the one described in figure 3(a) where a three electrode configuration was used. Although the values of the photocurrents measured in the two electrode configuration are six times smaller, the photocurrent transient shapes are identical to those measured in the three electrode configuration. This demonstrates that the absence of the QRE in the setup influences only the photocurrent flowing through the electrochemical cell but does not influence the electrochemical processes occurring under illumination. In this case the significance of the applied potential can be evidenced.

The behaviour of the PBDTTT-c:PCBM bulk heterojunction was studied using two electrode configuration in the dark and under laser illumination. The resulting photocurrent transients are plotted in red in the part (a) of figure 4. It can be noticed that the shape and the value of photocurrent transients are similar to the ones measured in the three electrode configuration. After starting the illumination, a strong negative peak with a minimum at −10 *μ*A cm^−2^ followed by a decrease of the negative photocurrent up to −2.5 *μ*A cm^−2^ can be observed. As mentioned before, this behaviour can be explained by the electrochemical instability of PCBM. Interestingly, in the following dark sequence, only a small peak due to the discharging of the BHJ/electrolyte interface can be observed in comparison with the three electrode configuration. Within the next illumination sequences the degradation can be again observed resulting in the smaller detected photocurrent. In the cell having three electrodes, the effective WE-CE potential present during the photoelectrochemical characterization can be drastically different from the one measured between the WE and QRE using the potentiostat.

The photocurrent observed with the two electrode configuration using laser illumination was compared with the one measured under white light irradiation. The measured photocurrent transients of the pristine PBDTTT-c (plotted in black) and PBDTTT-c:PCBM mixture (plotted in red) are presented in figure 4(b). In both cases, the white light irradiation of the pristine polymer and its mixture with the acceptor only slightly influences the photoelectrochemical response. Although the shape of the photocurrent transients is identical to the one obtained in the three electrode configuration, a small difference can be observed in the value of the measured photocurrent. The pristine polymer photocurrent reaches values close to −2 *μ*A cm^−2^ (later increasing) while for the PBDTTT-c:PCBM mixture the measured photocurrent was as low as −7 *μ*A cm^−2^. The reason of this behaviour can again be related to the energetic levels alignment.

**Figure 5.**
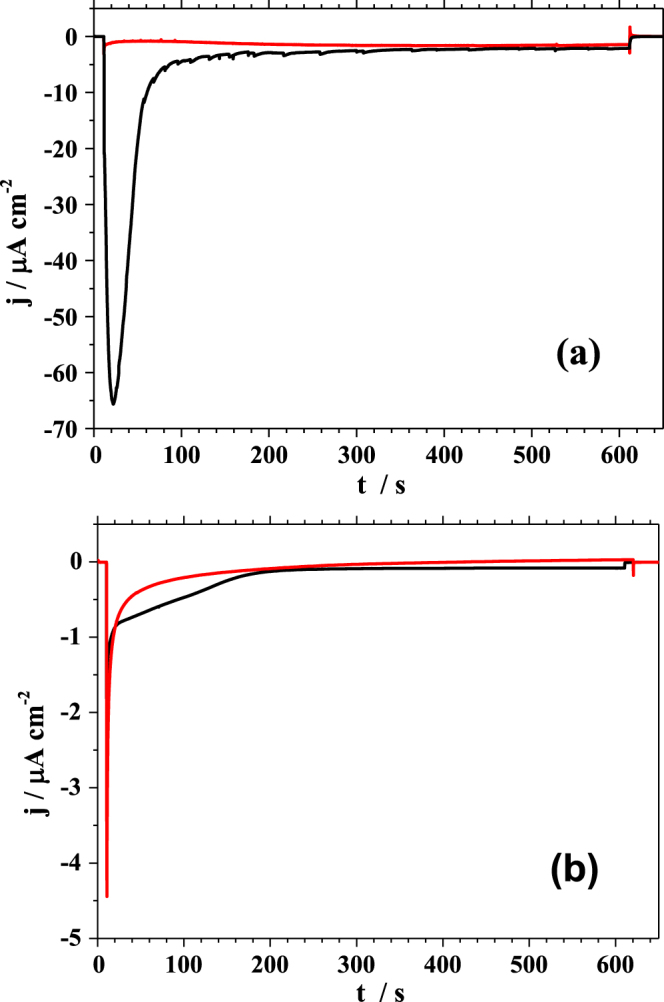
Current transients measured within 600 s during illumination with the red laser light (a) and white light (b). The measurements were performed in the three electrode configuration, on pristine PBDTTT-c (black line) and PBDTTT-c:PCBM (red line).

The stability of the photovoltaic effect for both PBDTTT-c and PBDTTT-c:PCBM was studied during longer photoelectrochemical measurements using the PE-SDCM. In this experiment both materials contacted with 0.1 M TBAPF_6_ dissolved in PC, were illuminated either with a single wavelength laser or with white light. During irradiation, 600 s photocurrent transients were measured. For a correct electrochemical characterization the measurements were performed using the three electrode configuration of PE-SDCM and the results are presented in figure 5.

In part (a) of figure 5 the effect of 600 s irradiation with laser light is presented. During the first 10 s of the experiment the PBDTTT-c (plotted in black) was kept in the dark and only background current was measured. After opening the shutter for radiation exposure a peak in the photocurrent can be observed within the next 100 s. The value and the shape of the photocurrent transient follow the behaviour of the photocurrent transients presented in figure 3. The photocurrent peak value reaches −65 *μ*A cm^−2^ after approximately 25 s. This matches the previous results of the experiment were dark/illumination sequences were used if one visualizes the envelope of the curves presented in figure 3(a). After another 100 s a photocurrent decrease is observed due to the degradation process and finally a negative photocurrent plateau at −6 *μ*A cm^−2^ is reached. A similar characterization was performed for the BHJ. The resulting photocurrent transient measured during 600 s of irradiation with single wavelength is plotted in red in part (a) of figure 5. The value and the shape of the photocurrent measured for BHJ in contact with electrolyte also visualizes the envelope of the corresponding curves presented in figure 3. A photocurrent decrease in the first second is observed which is followed by a continuous decrease up to a plateau positioned at −1.4 *μ*A cm^−2^. This behaviour of the negative photocurrent can also be attributed to the photodegradation of the PCBM.

The photocurrent stability measurements under laser irradiation performed for PBDTTT-c and PBDTTT-c:PCBM using the three electrode configuration for the PE-SDCM are compared with the corresponding curves detected when white light was used. The resulting photocurrent transients are plotted in figure 5(b). The photo-response of the pristine PBDTTT-c is plotted in black. Similar to the previous case of laser illumination, the photocurrent transients under white light irradiation follow the envelope of the corresponding photocurrent transients presented in black in figure 3(b). The photocurrent value obtained has a minimum of −3 *μ*A cm^−2^ and decreases reaching a plateau at −0.5 *μ*A cm^−2^ after 300 s. An identical situation can be observed for the case of PBDTTT-c:PCBM. The irradiation with white light results in a peak with a value of −4.5 *μ*A cm^−2^ which decreases within the first 100 s of the experiment. The final photocurrent measured after 600 s of irradiation is very low and can be considered as background.

**Figure 6.**
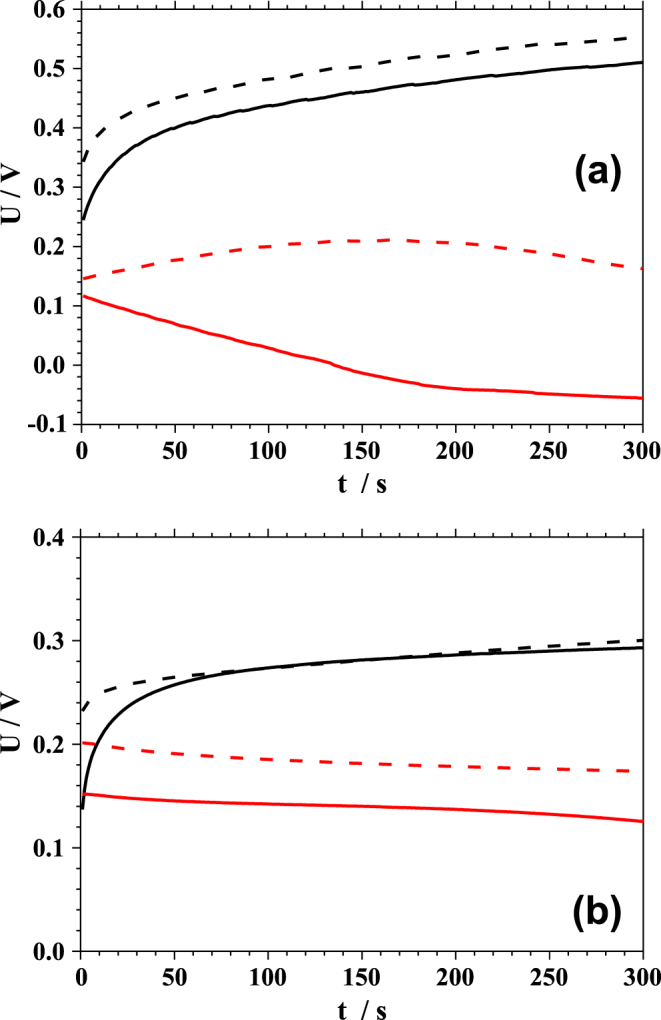
Potential transients measured within 300 s during illumination with the 635 nm laser light (a) and white light (b). The measurements were performed in three electrode configuration (solid line) and two electrode configuration (dashed line) for pristine PBDTTT-c (black line) and PBDTTT-c:PCBM (red line).

Beside the information about the current generated under illumination, the usual photovoltaic characterization also describes the behaviour of the energetic levels alignment. In the solar cells the open circuit potential is especially important since it describes the maximum potential generated when no current is flowing. The value of the open circuit potential is affected by all materials building the cell. The influence of the irradiation as well as measurement configuration on the open circuit potential of PBDTTT-c and PBDTTT-c:PCBM is studied. The resulting potential transients are presented in figure 6. The photovoltages measured in the three electrode configuration of the PE-SDCM for the pristine PBDTTT-c are plotted in black while those obtained for the BHJ are shown in red. The corresponding transients measured in the two electrode configuration are plotted using dashed lines.

The photovoltage measured during irradiation with laser light is presented in part (a) of figure 6. For pristine PBDTTT-c (plotted in solid, black) at first a potential of 0.24 V vs Ag/AgCl was measured. A continuous increase in the potential can be observed as a function of time. This increase can be explained by the continuous charging of the PBDTTT-c layer. After 300 s of laser illumination, the photovoltage reaches 0.51 V vs Ag/AgCl. A similar experiment was performed in the two electrode configuration (plotted dashed, black). The shape of the photovoltage transient in this case is identical to that obtained during measurement in the three-electrode configuration. However, the values are higher for each measured point by approximately 50 mV. In this way the effective potential between the WE and CE is directly probed and its increase can be related to the potential drop in the electrolyte and the work function of the CE.

Using laser light irradiation the photovoltage transients were also measured for PBDTTT-c:PCBM and the results are plotted in red in figure 6(a). The measurements were again performed in two (dashed line) and three (solid line) electrode configuration. As can be noticed for the PBDTTT-c:PCBM, a strong difference is found depending on the used configuration. In the three electrode configuration (where the photovoltage is referred to the QRE) a photovoltage decrease with time can be observed. After starting the radiation exposure the measured photovoltage was 0.12 V vs Ag/AgCl and 300 s later decreased to −0.04 V vs Ag/AgCl. This change in the polarization of the photovoltage can be related to the charging of the PCBM. The photovoltage of PBDTTT-c:PCBM measured in the two electrode configuration shows an opposite behaviour. After opening the shutter, the value of the photovoltage was found to be 0.15 V, similar to the one obtained in the three electrode configuration. During the 300 s illumination sequence this value slightly increases. As previously discussed, the measured photovoltage is strongly dependent from the effective potential between the WE and CE.

Finally the effect of illumination on the photovoltage transients in PBDTTT-c (plotted in black) and PBDTTT-c:PCBM (plotted in red) were characterized under white light irradiation in the two (dashed line) and three electrode (full line) configuration. The resulting photovoltage transients are presented in figure 6(b). The photovoltage measured for the pristine PBDTTT-c in the three electrode configuration shows a similar behaviour to those obtained during laser illumination. After opening the shutter the value of the photovoltage was measured at 0.14 V vs Ag/AgCl and an increase during the measurement period up to almost 0.3 V vs Ag/AgCl. As previously described, this increase results from photocharging of the PBDTTT-c layer. Similar to the photocurrent behaviour, the value of the photovoltage is much smaller during illumination with white light then the one obtained during laser illumination. Since in the electrochemical cell the photovoltage corresponds to the difference between the oxidation potential of the material and the redox potential of the electrolyte, the change in the potential can be related to a decrease of the band position in the gap. This effect can be seen, since during illumination of the PBDTTT-c with a white light all absorption energies are probed. During the illumination with the laser irradiation only the lowest optical transition is probed. When comparing the results of the photovoltage measured in two electrode configuration (dashed line) or in three electrode configuration (solid line), several observations can be made. Although the shapes of both curves are similar, within the first 80 s of measurement time differences in the measured values can be seen. The photovoltage measured in the two-electrode configuration just after starting the illumination is by 80 mV higher and with time this difference decreases. Using white light irradiation the photovoltage transients were additionally measured for PBDTTT-c:PCBM and the results are plotted in red in figure 6(b). In the three electrode configuration (solid lines) the photovoltage was first measured as 0.15 V vs Ag/AgCl. Within 300 s the potential slightly decreases with time. A similar experiment was performed in the two electrode configuration (plotted dashed, red). The shape of the photovoltage transient is identical to that obtained during measurement in the three-electrode configuration with the values higher by 50 mV at each measurement point.

## 4. Conclusions

The photoelectrochemical properties of the PBDTTT-c as well as PBDTTT-c:PCBM were studied using PE-SDCM. The possibility of addressing small areas on the surface of the working electrode combined with the advantage of using low electrolyte volumes (<1 ml) have been exploited in this study. Characterization was performed under illumination with single wavelength light (633 nm) fitting to the maximum absorption of the polymer and compared with results obtained during illumination with white light. The behaviour of the photocurrent was characterized using dark/illumination sequences and compared with the longer term illumination. It was found that an addition of the PCBM strongly decreases the photocurrent due to the photoelectrochemical degradation of the acceptor. Furthermore the photocurrent is strongly dependent on the illumination condition. Using white light not only influences the photocurrent value but also the photocurrent behaviour. Finally the photovoltage transients were studied. It was found that the photoinduced potential is strongly dependent from the configuration (three or two electrode) and illumination conditions.

## References

[C1] Hoppe H, Sariciftci N S (2004). J. Mat. Res..

[C2] Park S H, Roy A, Beaupre S, Cho S, Coates N, Moon J S, Moses D, Leclerc M, Lee K, Heeger A J (2009). Nat. Photon..

[C3] Irimia-Vladu M (2012). Adv. Mat..

[C4] Wang E, Hou L, Wang Z, Hellstroem S, Zhang F, Inganas O, Andersson M R (2010). Adv. Mat..

[C5] Kaltenbrunner M, White M S, Glowacki E D, Sekitani T, Someya T, Sariciftci N S, Bauer S (2012). Nat. Commun..

[C6] White M S (2013). Nat. Photon..

[C7] Cheng Y J, Yang S H, Hsu C S (2009). Chem. Rev..

[C8] Service R F (2011). Science.

[C9] Abou-Ras D, Cahen D, Nou R, Unold T, Green M A, Emery K, Hishikawa Y, Warta W, Dunlop E D (2012). Prog. Photovolt. Res. Appl..

[C10] Chen H Y, Hou J, Zhang S, Liang Y, Yang G, Yang Y, Yu L, Wu Y, Li G (2009). Nat. Photon..

[C11] Hou J, Chen H Y, Zhang S, Chen R I, Yang Y, Wu Y, Li G (2009). J. Am. Chem. Soc..

[C12] Huo L, Zhang S, Guo X, Xu F, Li Y, Hou J (2011). Angew. Chem..

[C13] He Y, Chen C, Richard E, Dou L, Wu Y, Li G, Yang Y (2012). J. Mater. Chem..

[C14] Zhang Z G, Li H, Qi Z, Jin Z, Liu G, Hou J, Li Y, Wang J (2013). Appl. Phys. Lett..

[C15] Kang T E, Cho H H, Cho C H, Kim K H, Kang H, Lee M, Lee S, Kim B, Im C, Kim B J (2013). ACS Appl. Mater. Inter..

[C16] Ohkita H, Ito S (2011). Polym..

[C17] Kyu An T, Eon Park C, Sung Chung D (2013). Appl. Phys. Lett..

[C18] Hassel A W, Lohrengel M M (1997). Electrochim. Acta.

[C19] Mardare A I, Hassel A W (2009). Rev. Sci. Instr..

[C20] Kollender J P, Mardare A I, Hassel A W (2013). Chem. Phys. Phys. Chem..

[C21] Gasiorowski J, Mardare A I, Sariciftci N S, Hassel A W (2013). J. Electroanal. Chem..

[C22] Hassel A W, Fushimi K, Seo M (1999). Electrochem. Comm..

[C23] Gasiorowski J, Mardare A I, Sariciftci N S, Hassel A W (2013). Electrochim. Acta.

[C24] Yu P Y, Cardona M (2010). Fundamentals of Semiconductors. Physics and Materials Properties.

[C25] Azzam R M A, Bashara N M (1987). Ellipsometry and Polarized Light.

[C26] Gasiorowski J, Menon R, Hingerl K, Dachev M, Sariciftci N S (2013). Thin Solid Films.

[C27] Gasiorowski J, Hingerl K, Menon R, Plach T, Neugebauer H, Wiesauer K, Yumusak C, Sariciftci N S (2013). J. Phys. Chem. C.

[C28] Scharber M C, Sariciftci N S (2013). Progr. Polym. Sci..

[C29] Blom P W M, Mihailetchi V D, Koster L J A, Markov D E (2007). Adv. Mater..

[C30] Brabec C J, Gowrisanker S, Halls J J M, Laird D, Jia S, Williams S P (2010). Adv. Mater..

[C31] van Duren J K J, Yang X, Loos J, Bulle-Lieuwma C W T, Sieval A B, Hummelen J C, Janssen R A J (2004). Adv. Funct. Mater..

[C32] Graetzel M (2003). J. Photochem. Photobiol. C: Photochem. Rev..

